# Threats to the conservation of the vulnerable giant anteater (*Myrmecophaga tridactyla*) in the Cerrado biome: a retrospective survey

**DOI:** 10.29374/2527-2179.bjvm001023

**Published:** 2023-08-21

**Authors:** Nathana Beatriz Martins, Nataly Nogueira Ribeiro Pinto, Tainara Santana Galvão da Silva, Aline Santana da Hora

**Affiliations:** 1 Veterinarian, Resident. Programa de Residência em Medicina Veterinária - Medicina de Animais Selvagens. Universidade Federal de Uberlândia (UFU), Uberlândia, MG, Brazil; 2 Veterinarian, DSc. UFU, Uberlândia, MG, Brazil

**Keywords:** habitat fragmentation, roadkill, Xenarthra, atropelamentos, fragmentação de habitat, Xenarthra

## Abstract

In this study, we conducted a retrospective survey of 63 giant anteaters (*Myrmecophaga tridactyla*) using the Federal University of Uberlândia, Minas Gerais State, Brazil as reference site for wild animals. We analyzed the clinical records of 63 animals from January 2016 to February 2020. The information obtained included the location where the anteater was found, the reason for rescue, estimated life stage, gender, weight, general condition of the animal, clinical signs, diagnosis, and destination. Of the 63 animals, 30.15%, (n = 19/63) were found in rural areas, 25.40% (n = 16/63) in urban areas, and 22.22% (n = 14/63) near highways. The main reason for rescue was run-over accidents (n = 18/63, 28.60%). Regarding life stage distribution, 27% (n = 17/63) were cubs, 25.40% (n = 16/63) were adolescent, and 41.26% (n = 26/63) were adults. There was a higher frequency of females (n = 35/63, 56%), and three (9%) of them were pregnant or had cubs. For injury evaluation, three of the 63 giant anteaters were dead on arrival at the rehabilitation site; therefore, we excluded them from this aspect of the study. Of the 60 remaining anteaters, only 13.33% (n = 8/60) of the animals were healthy upon physical examination.The most common condition was traumatic brain injury (n = 32/60 53.33%), followed by fractures (n = 23/60, 38.33%), neonate triad (n = 15/60, 25%), and abrasions (n = 15/60, 25%). The animals presented a high mortality rate (n = 39/60, 65%). The low number of giant anteaters reintroduced to their natural habitat and the high mortality rate of animals sent to rehabilitation centers show that the protection of giant anteaters is important to reduce the number of these animals sent to rehabilitation centers.

## Resumo

Foi realizado um levantamento retrospectivo de 63 tamanduás-bandeira (*Myrmecophaga tridactyla*) atendidos em um centro de animais selvagens referência na Universidade Federal de Uberlândia, Minas Gerais, Brasil. Foram analisados registros clínicos de 63 animais de janeiro de 2016 a fevereiro de 2020. As informações coletadas foram: local onde foi encontrado, motivo do resgate, idade estimada, sexo, peso, estado geral do animal, sinais clínicos, diagnóstico e destinação. Os animais foram encontrados em áreas rurais (30.15%, n = 19/63), áreas urbanas (25.40%, n = 16/63) e próximo a rodovias (22.22%, n = 14/63). O principal motivo do resgate foram os atropelamentos (28.60%, n = 18/63). A faixa etária foi de 27% (n = 17/63) filhotes, 25.40% (n = 16/63) jovens e 41.26% (n = 26/63) adultos. Encontrou-se maior frequência de fêmeas (56%, n = 35/63), sendo que três (9%) estavam prenhes ou com os filhotes nas costas. Apenas 13.33% (n = 8/60) dos animais apresentavam-se saudáveis ao exame físico. A condição mais comum foi traumatismo cranioencefálico (53,33%, n = 32/60), seguida por fraturas (38.33%, n = 23/60), tríade neonatal (25%, n = 15/60) e escoriações (25%, n = 15/60). Os animais apresentaram alta taxa de mortalidade (65%, n = 39/60). O baixo número de tamanduás reintroduzidos em seu habitat natural e a alta taxa de mortalidade de animais encaminhados para centros de reabilitação, mostram a importância de medidas que os protejam a ponto de reduzir o número de animais encaminhados para esses centros.

## Introduction

The giant anteater (*Myrmecophaga tridactyla* Linnaeus, 1758) is a placental mammal of the Xenarthra superorder and is considered a species vulnerable to extinction according to the International Union for Conservation of Nature (IUCN) (Superina et al., 2010). The main threat to these species is anthropogenic interference, especially habitat destruction and fragmentation, burning, run-over accidents, dog attacks, and killing due to retaliation or superstition (Miranda et al., 2015). Giant anteaters have physiological characteristics that contribute to their vulnerability, such as low mobility and poor vision, which results in them often becoming victims of run-over accidents on highways with a high mortality rate (Diniz & Brito, 2015).

Cerrado is the second largest biome in Brazil and a hotspot for biodiversity conservation because this tropical savannah hosts approximately 4,800 species of plants and vertebrates (Strassburg et al., 2017). However, this biome is threatened by the continuous conversion of the native vegetation into cultivable areas, resulting in a loss of 48% (88 Mha) of its native vegetation; only 19.8% remains undisturbed (Strassburg et al., 2017).

The Cerrado biome covers approximately 2 million km^2^ of Central Brazil, representing approximately 23% of the land surface of the country. The Cerrado region extends from the margin of the Amazonia Forest to outlying areas in the southern states of São Paulo and Paraná, occupying more than 20º of latitude and has an altitudinal range of 0 (sea-level) to 1,800 m (Ratter et al., 1997).

The climate is seasonal, wet from October to March and dry from April to September, and mild year-round temperatures ranging from 22 to 27 ºC. The average annual rainfall is 1,500 mm (Haridasan, 1982). More than half of the 2 million km^2^ has been transformed into pasture, cash crop agriculture, and other uses in the last 35 years (Klink & Machado, 2005).

Therefore, a retrospective survey of giant anteaters, with data obtained from a reference for wild animals in the Cerrado biome was conducted to expand our understanding about the different population aspects of this species and their vulnerability.

## Materials and methods

A retrospective study was conducted based on an evaluation of 63 clinical records of giant anteaters obtained from the Federal University of Uberlândia, Minas Gerais State, Brazil, which serves several municipalities in the Cerrado biome in the states of Minas Gerais and Goiás, Brazil.

The clinical records of individual animals were analyzed from January 2016 to February 2020. Data were collected and tabulated; this data included information on the location where the anteater was found, the reason for rescue, date of arrival, estimated life stage (cub: weight up to 6 kg; adolescent: 6–25 kg, and adult: over 25 kg), gender, weight, general condition of the animal, clinical signs, diagnosis, and destination of the animal. Subjective parameters were considered for evaluation and included dehydration (mild [1–5%], moderate [6–11%], and severe [≥ 12%]) and body condition score, which was rated using a 5-point system (1 = emaciated; 2 = too thin; 3 = ideal weight; 4 = too fat; 5 = obese) (Freeman et al., 2011). The results were analyzed and expressed by absolute numbers and percentage (%).

## Results

**Figure 1:**
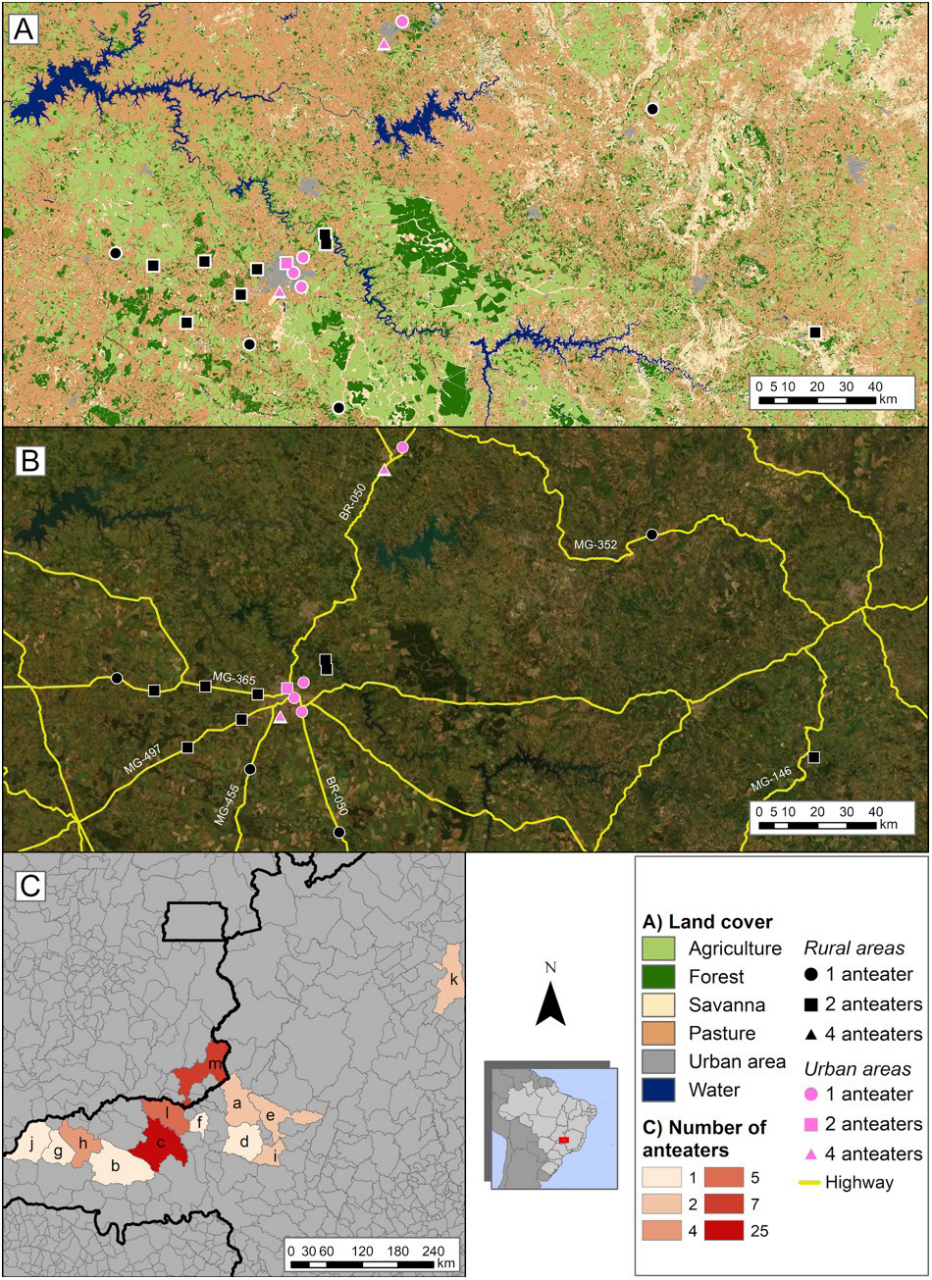
Locations of rescued giant anteaters (*Myrmecophaga tridactyla*) obtained from a reference site for wild animals in the Cerrado biome from 2016 to 2020. (A) Animal rescue locations and land type. (B) Rescue locations on highways. (C) Municipalities in Minas Gerais and Goiás where the animals were rescued: a = Coromandel; b = Prata; c = Uberlândia; d = Patrocínio; e = Patos de Minas; f = Estrela do Sul; g = Gurinhatã; h = Ituiutaba; i = Serra do Salitre; j = Santa Vitória; k = Montes Claros; l = Araguari; m = Catalão.

Sixty-three giant anteaters were observed at Federal University of Uberlândia during the 48 months surveyed. Of the 63, 19 (30.15%) were found in rural areas followed by 16 (25.40%) in urban areas and 14 (22.22%) near highways (Figure 1). The rescue locations of 14 (22.22%) giant anteaters were not recorded in the clinical records. Run-over accidents were the main reason these animals needed rescue and rehabilitation (Table 1).

**Table 1:** Reasons for the rescue of giant anteaters (*Myrmecophaga tridactyla*) and their relative distribution.

Reason for rescue	Number of animals	Percentage (%)
Run-over accidents	18	28.60
Animal found on a public road	10	15.90
Cub found alone	9	14.28
Not informed	8	12.7
Found lying down	4	6.34
Dog attack	4	6.34
Found injured	3	4.76
Referred by other institutions	3	1.59
Found on a tractor engine	1	1.59
Found in a lagoon after escaping domestic dogs	1	1.59
Cub found on its dead mother's back	1	1.59
Under human care	1	28.60
*Total*	63	100

Data obtained from the Federal University of Uberlândia, in Uberlândia, Minas Gerais State, Brazil, from 2016 to 2020.

Three of the giant anteaters were dead on arrival at the site. Of the 63 giant anteaters 35 (58.3%) were female and 26 (43.4%) were adults (over 25 kg in weight). The data on gender and estimated life stage of the animals are shown in Table 2.

**Table 2:** Distribution of sex and estimated age range of giant anteaters (*Myrmecophaga tridactyla*).

	Life stage				Gender		
	**Cub**	**Young**	**Adult**	**NI**	**M**	**F**	**NI**
Number of animals	17	16	26	4	22	35	6
Percentage (%)	27.0	25.4	41.26	6.34	35	56	9
*Total*		63			63		

Data obtained from the Federal University of Uberlândia, in Uberlândia, Minas Gerais State, Brazil from 2016 to 2020.

NI = not informed; M = male; F = female.

Eight (13.3%) animals were healthy upon physical examination. Apathy and different degrees of dehydration were the most frequently observed clinical signs (Table 3). Three animals were dead on arrival at the site and were excluded from the data (n = 60). Of the remaining 60 cases, the most common condition was traumatic brain injury (32, 53.3%), followed by fractures (23, 38.33%), neonate triad (15, 25.0%), abrasions (15, 25.0%), myopathy (3, 5.0%), and burns (1, 1.6%).

**Table 3:** Distribution of clinical signs and parameters obtained from the physical examinations of rescued giant anteaters (*Myrmecophaga tridactyla*).

Clinical signs	Number of observations per animal	Percentage (%)
Mild dehydration	7	3.4
Moderate dehydration	9	4.4
Severe dehydration	11	5.5
Normal body condition score	17	8.4
Regular body condition score	2	0.9
Poor body condition score	11	5.5
Alert behavior	13	6.4
Active behavior	12	5.9
Aggressive behavior	3	1.4
Apathic behavior	36	17.9
Paresis	2	0.9
Muscle atrophy	1	0.4
Myoclonus	1	0.4
Hypoglycemia	5	2.4
Hypothermia	5	2.4
Hyperthermia	2	0.9
Hypotension	1	0.4
Anorexia	5	2.4
Absence of lingual tone	2	0.9
Tongue eversion	5	2.4
Mucosal congestion	3	1.4
Nasal clot	2	0.9
Nasal discharge	1	0.4
Dyspnea	1	0.4
Pulmonary crackling	1	0.4
Pulmonary edema	2	0.9
Thorax perforation	1	0.4
Eyelid laceration	1	0.4
Conjunctivitis	2	0.9
Corneal ulcer	1	0.4
Eye protrusion	2	0.9
Diarrhea	3	1.4
Feces impaction	2	0.9
Melena	1	0.4
Intestinal parasites	3	1.4
Parasites in stomach	2	0.9
Ixodidiosis	10	4.9
Tungiasis	4	1.9
Myiasis	1	0.4
Pressure bruises	1	0.4
Abdominal distention	2	0.9
Lactation	3	1.4
Alopecia	2	0.9
*Total*	201	

Data obtained from the Federal University of Uberlândia, in Uberlândia, Minas Gerais State, Brazil, at the time of arrival from 2016 to 2020.

Note: Data from clinical signs of 60 giant anteaters were analyzed as three individuals were dead on arrival at the rehabilitation center.

The clinical signs presented by the animals diagnosed with traumatic brain injury (TBI) are shown in Table 4. Bone disorders consisted of seven polytraumatized individuals (30.43%), four humerus fractures (17.39%), two radius and ulna fractures (8.69%), two femur fractures (8.69%), one multiple phalanx fracture (4.34%), one tibial fracture (4.34%), one complete lumbar fracture (4.34%), one pelvis fracture (4.34%), one Monteggia fracture type 1 (4.34%), one radius fracture (4.34%), one nasal bone fracture (4.34%), and one fracture that was not specified in the clinical record (4.34%).

**Table 4:** Distribution of neurological clinical signs in giant anteaters (*Myrmecophaga tridactyla*) diagnosed with a traumatic brain injury (n = 32).

Neurological clinical signs	Number of observations per animal	Percentage (%)
Apathy	12	12.2
Prostration	7	7.1
Stupor	14	14.2
Coma	11	11.2
Nystagmus	16	16.3
Anisocoria	10	10.2
Opisthotonos	4	4.0
Convulsions	8	8.1
Ataxia	2	2.0
Incoordination	3	3.0
Paresis	2	2.0
Paralysis	1	1.0
Absent direct and consensual pupillary reflex	5	5.1
Superficial pain absent	2	2.0
Deep pain absent	1	1.0
*Total*	98	

Data obtained from the Federal University of Uberlândia, in Uberlândia, Minas Gerais State, Brazil, from 2016 to 2020.

Note: Data from clinical signs of 32 giant anteaters were analyzed.

The animals presented a high mortality rate (39/60, 65%). Of the surviving animals, only three healthy individuals found in urban areas (5%) were released near the place where they were found. Another three healthy individuals (5%) were sent to other institutions to be kept in captivity, as two were cubs and one was an adolescent that had been under human care for a long period.

However, seven non-healthy animals were reintroduced into the wild (12%), of which five were adults and two were juveniles. Five of these seven individuals were run-over accident victims and presented TBI (3/5) and/or fractures in the radius and ulna (1/5), pelvis (1/5) and Monteggia (1/5). One animal was found in an urban area presenting TBI, and another had no history, only lesions in the left ventral cervical region. This demonstrated the highest survival rate of young and adult animals.

Eight (13.33%) of the giant anteaters died but were classified as healthy. These were all cubs that were found alone in rural areas, on public roads, or on their deceased mother's back. All cubs were classified as alert and active with mild dehydration when they arrived at the site, excluding one cub that was apathetic. These results demonstrate the high mortality of cubs.

## Discussion

The giant anteater is a symbolic species of the Cerrado biome. However, there are few scientific studies on this species. The present study was conducted on giant anteaters from the Cerrado biome and presents the main threats to this species. Our findings may contribute to the efforts to conserve giant anteaters.

Run-over accidents was the main reason giant anteaters needed rescue; this corroborates data from screening and rehabilitation centers in Brazil. Their slow movement and poor vision increases the occurrence of run-over accidents, as well as the lack of measures for fauna protection near roads and to reduce high-speed traffic (Miranda et al., 2015). Moreover, Minas Gerais has the longest road network in Brazil, which demonstrates the significant fragmentation of the natural habitat and increases the probability of road accidents involving many species including the giant anteater.

The giant anteater is the mammal most victimized by road accidents. A total of 51 individuals were victims in road accidents according to a collaborative data collection system, which obtains data from people who report road accidents involving wild animals. This data collection system showed 387 hit-and-run incidents involving anteaters since 2014. Few studies in Brazil characterize the negative effects of road accidents with wild fauna, despite it being one of the main negative impacts on giant anteater populations (Collevatti et al., 2007).

The number of animals rescued on highways in the study period was 14 (22.22%). The roads where the most animals were rescued were the BR-050 highway, followed by the MG-365 highway. The number of animals killed on Brazilian roads is more greater on highways with high-traffic roads that cut through areas with abundant fauna and flora.

The data presented in this study confirms those from surveys of hit-and-runs of Xenarthra species on highways in the Cerrado biome region. More than 90.0% of these surveys included Xenarthra among the top five species with the most reported incidents; 33.3% reported that Xenarthra species represent more than 50% of the hit-and-runs recorded and another 33.3% of the surveys reported that other species belonging to the Pilosa order were involved in more than 25.0% of the hit-and-runs recorded (Ribeiro et al., 2017). This data reinforces the idea that run-over accidents are one of the greatest threats to populations of Xenarthra in Brazil.

Destruction and fragmentation of habitats are also important causes for the decrease in giant anteater populations (Collevatti et al., 2007). In the present study, most giant anteaters (19/63, 30.15%) were found in rural areas. Most locations where the animals were rescued had little to no preserved areas (forests and savannas) (Figure 1A). No animals were rescued in native areas, 11.76% (4/34) of the animals were rescued in pastures close to native areas, 11.76% (4/34) were rescued in pastures close to agricultural land and native areas, and 2.94% (1/34) in agricultural land near native areas. The data showed that the animals were typically found in urban and rural areas, indicating habitat loss due to the scarcity of forest areas.

The giant anteater uses agricultural land and pasture areas as a dispersion region or as part of its living area (Vynne et al., 2011). However, native vegetation fragments are required for the appropriate development of physiological functions of the giant anteaters, which is mainly thermal regulation, because the they are sensitive to the high temperatures that occur during the day (Miranda et al., 2015). In the present study, eight animals (23.5%) were rescued close to poorly preserved vegetation areas.

Some giant anteaters were rescued in urban areas (25.40%). Their occurrence in these areas is probably due to the destruction and fragmentation of their habitat and consequent migration to urban areas, which is shown by the numerous healthy animals (84.0%) found in urban areas. This reinforces the hypothesis that the animals migrate to urban areas because of fragmentation and destruction of their natural habitat.

Regarding sex, females were more frequently found, which included pregnant females and females with cubs, which is a warning for conservation efforts. This result is consistent with other studies, which reported high rates of females involved in run-over accidents, decreasing giant anteater populations in their habitat. This is further aggravated by the fact that giant anteaters have a long gestation and parental care periods and females only have one cub per year (Miranda et al., 2015).

The numerous giant anteater cubs orphaned by run-over accidents and other causes is also an important factor for the protection of the species, because maintaining these individuals in captivity with artificial breastfeeding is difficult and results in high mortality rates. Few studies have investigated the nutritional requirements of anteaters (Miranda et al., 2015). To care for animals of the Xenarthra superorder, specific anatomical, dietary, nutritional, and behavioral knowledge is required, and these requirements are not yet well understood. This problem, combined with numerous rescued cubs is another threat to the conservation of the species.

No surveys evaluating the clinical conditions of giant anteaters sent to wildlife screening and rehabilitation centers were found. However, data compiled on collared anteaters (*Tamandua tetradactyla*) over 13 years were found for a region in São Paulo (Southeast region of Brazil), which presented run-over accidents (17/43, 37.0%) as the most frequent cause for the rescue and rehabilitation of these animals (Bernegossi et al., 2018), as confirmed by the present study in which run-over accidents were responsible for 28.60% of giant anteaters needing rescue.

That same study showed that the reintroduction rate of collared anteaters to the wild was 37.0% (17/43) (Bernegossi et al., 2018), whereas the present study found that 28.3% (17/60) of the giant anteaters were reintroduced to their natural habitat. The collared anteaters (35.0%, 16/43) also presented a lower mortality rate when compared to that of the giant anteaters in the present study (39/60, 65%). Injuries resulting from trauma were the main reason for the death of giant anteaters (63%), and 39.4% of the deaths were cubs because orphaned giant anteaters are commonly found in cities within regions where the species occurs. The mothers of these cubs are typically killed by burning, run-over accidents, and hunting (Miranda et al., 2015).

## Conclusions

The small number of giant anteaters reintroduced to their natural habitat and the high mortality rate of those that are rescued and sent to screening and rehabilitation centers shows that measures or policies to protect giant anteaters are required. The data presented in this study show that giant anteaters are constant victims of run-over accidents on highways. Moreover, the habitats of these animals have been fragmented and destroyed, forcing them to migrate to rural and urban areas occupied by humans, resulting in numerous deaths, injuries, and orphaned animals. This retrospective study highlights the need for conservation programs to reduce the mortality rate of these animals owing to anthropogenic activities.
